# Tunable White Light Emission from Transparent Nanophosphor Films Embedding Perovskite Lead Halide Nanostructures

**DOI:** 10.1021/acsami.4c22044

**Published:** 2025-03-19

**Authors:** José María Viaña, Carlos Romero-Pérez, Mauricio E. Calvo, Gabriel Lozano, Hernán Míguez

**Affiliations:** Instituto de Ciencia de Materiales de Sevilla, Consejo Superior de Investigaciones Científicas-Universidad de Sevilla, Calle Américo Vespucio 49, Sevilla 41092, Spain

**Keywords:** ABX_3_ CsPbBr_3_, Cs_4_PbBr_6_, perovskite nanocrystals, nanophosphors, white emission

## Abstract

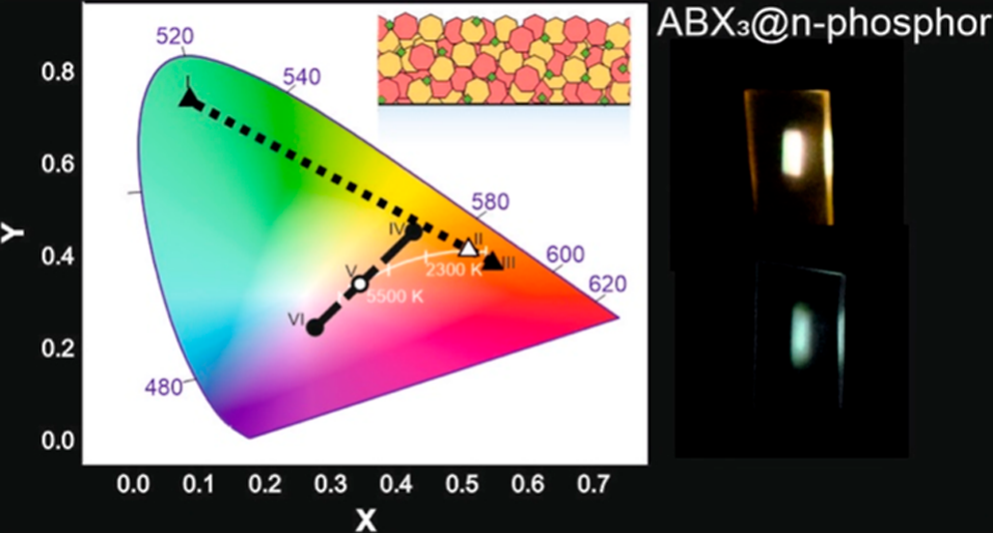

Exploring synergistic interactions between nanomaterials that can enhance their collective properties in ways that individual components cannot achieve represents an avenue for advancing beyond the current state of the art. This approach is particularly relevant in the context of ABX_3_ nanocrystals, where pursuing cooperation could help to overcome current challenges associated with light generation. Transparent photoluminescent coatings are developed by combining perovskite nanomaterials and porous scaffolds of high optical quality phosphor nanoparticles. Fine tuning of the spectral content of the emission is achieved with the photoexcitation wavelength, allowing the demonstration of white light emission with tunable hues.

## Introduction

Lead halide perovskite (ABX_3_) nanocrystals have burst onto the scene as luminescent nanomaterials, promising endless possibilities for a wide range of applications, including displays, solar concentrators or photodetectors.^1,2^ They exhibit outstanding properties such as bandgap tunability throughout the visible and high radiative carrier recombination, resulting in near-unit photoluminescence quantum yield (PLQY), and excellent chromatic purity.^3−6^ Nevertheless, it should be noted that emitter properties are not uniform across the visible, as it is difficult to demonstrate emission in the blue, and efficiency or stability in the red is rarely as high as in the green, challenging pure perovskite white light generation.^7^ The main synthetic approach to obtain perovskite nanocrystals (PNCs) is the colloidal route, either by hot injection^8−11^ or ligand-assisted reprecipitation.^12−15^ However, these methods require precise control of synthesis conditions, multiple purification steps, and significant amounts of different solvents, which is often cumbersome and not environmentally friendly.^16^ In addition, when perovskite quantum dot colloids are deposited as thin films, as is often required for any optical application, agglomeration and degradation of the particles occur, limiting their processability and compromising their properties.^17−19^

To overcome some of these issues, an alternative synthetic approach based on porous scaffolds made of nanoparticles of sub-100 nm size and regular shape has been developed. The pore network acts as nanometric-scale reactor, so that control over pore size, shape and connectivity ultimately determines the properties of the nanomaterials.^20,21^ As a result, a variety of ligand-free PNCs have been fabricated using porous thin films made of oxides of different compositions, such as SiO_2_ or Al_2_O_3_, as scaffolds.^22−26^ Interestingly, if the scaffolds are already supported in a substrate, PNCs can be directly processed as a thin film without additional preparation steps.^27−31^ This approach also enables the PNC film to be imbued with other functionalities inherited from the nanoparticles that compose the scaffold, opening avenues for the development of multifunctional composite materials that have barely been explored.

Phosphor nanoparticles, commonly referred to as nanophosphors, are chemically and thermally stable inorganic crystals doped with rare earth ions with excellent emission properties.^32,33^ It is worth noting that these materials form the basis for color conversion in solid-state lighting.^34,35^ Although perovskite materials have been combined with phosphor particles such as yttrium aluminum garnet (YAG) or potassium fluorosilicate (KSF) for color conversion coatings,^36−41^ there is no evidence of PNCs and nanophosphors being combined in a transparent thin film, nor any examples of nanophosphor scaffolds being used for PNC synthesis. The incompatibility between solvents and the different thermal processing prevents further development and integration of both nanomaterials.

In this work, we synthesize CsPbBr_3_ and Cs_4_PbBr_6_ nanocrystals, which emit green and blue light respectively, within porous films made of GdVO_4_:Eu^3+^ and GdVO_4_:Dy^3+^ nanoparticles. When excited with ultraviolet (UV) light, Eu^3+^-doped nanoparticles emit red light, while Dy^3+^-doped nanocrystals emit light in the blue and yellow regions of the visible spectrum. We optimize the deposition of the films to minimize light scattering and to achieve both transparency and efficient photoexcitation, resulting in bright emission. Furthermore, the shade of the emitted light from a given thin film can be continuously adjusted without altering the chemical composition of the emitters, through the gradual shift of the photoexcitation wavelength. As a result, the combination of perovskite and phosphor nanomaterials creates transparent coatings that emit white light with tunable hues ranging from warm (2300 K) to neutral (5500 K).

## Results and Discussion

### Perovskite Nanocrystals Embedded in Phosphor Nanoparticle Thin Films

**Figure 1:**
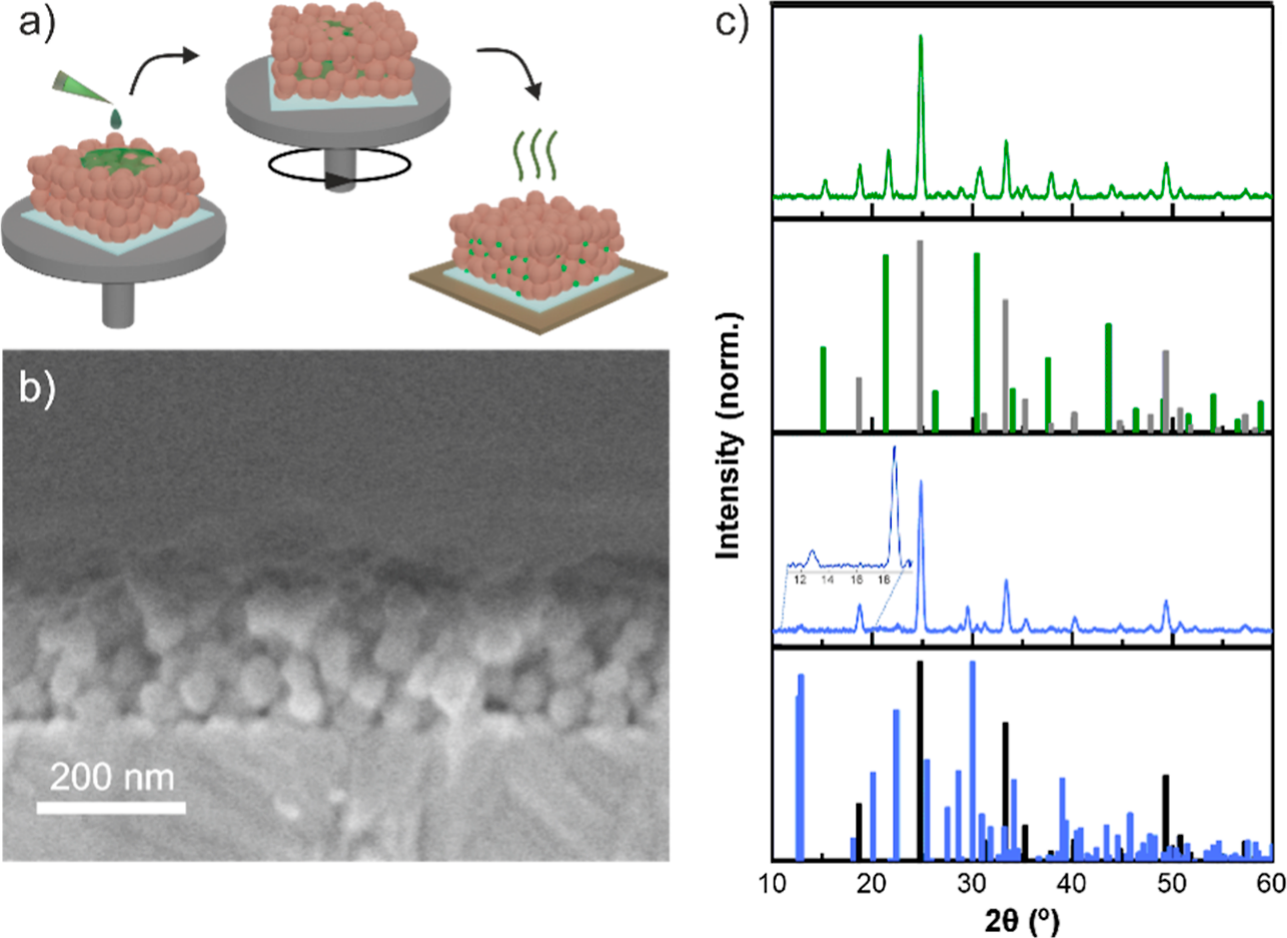
(a) Schematic of the preparation method (b) scanning electron microscope image of a cross-section of a GdVO_4_:Eu^3+^/Dy^3+^ nanoparticle film embedded with CsPbBr_3_ NCs. (c) X-ray diffraction patterns of CsPbBr_3_@GdVO_4_:Eu^3+^/Dy^3+^ (green curve) and Cs_4_PbBr_6_@GdVO_4_:Dy^3+^ (violet curve) films, along with reference patterns for comparison (PDF-01-086-0996 in black in bottom panel and gray in upper middle panel, PDF-01-084-0464 in green, and PDF-01-073-2478 in violet). The inset displays a closer look between 10 and 20°, so the most distinctive peaks of Cs_4_PbBr_6_ can be unambiguously observed.

In order to prevent light scattering and facilitate film processing, it is necessary for the phosphor nanoparticles to be small and colloidally stable in polar solvents.^42^ In order to achieve so, GdVO_4_ nanoparticles were synthesized via a solvothermal route, while two different rare earth ions (Dy and Eu) were employed as dopants to achieve GdVO_4_:Dy^3+^ and GdVO_4_:Eu^3+^ phosphor nanoparticles. Transparent phosphor thin films made of GdVO_4_:Dy^3+^ and a mixture of GdVO_4_:Dy^3+^ and GdVO_4_:Eu^3+^ nanoparticles were prepared by spin-coating using these nanoparticles as building blocks. This procedure is carried out under controlled conditions of speed and acceleration to attain films with uniform thickness.^44^ A mild heat treatment is then applied to remove any remaining solvent, which also facilitates the sequential deposition of additional layers. An additional annealing at 400 °C is then required to increase the PLQY of the phosphor nanoparticle film.^45^ After this treatment, the thickness of the phosphor scaffold was found to be approximately 300 nm. Figure S1 shows a top view of a thermally treated phosphor film, where porosity is clearly visible. In fact, spectroscopic techniques can be used to estimate the effective refractive index of the layers, which was found to be ∼1.4 in the visible, corresponding to ∼55% filling, leaving a porosity of ∼45%. Notice that although annealing at higher temperatures can improve further the photophysical properties of the nanophosphor films, it can also cause nanoparticle coalescence, leading to a reduction of porosity and an increase of the scattering, hence undermining their use as scaffolds.^38,46^ Sequentially, perovskite nanocrystals were synthesized within the film void network via precursor infiltration. Figure 1a depicts the synthesis of PNCs using a porous nanophosphor film as a scaffold. This step involves depositing a perovskite precursor solution on top of the phosphor thin film, followed by spin-coating, which causes the infiltration of reactants throughout the pore network while preventing the formation of a bulk perovskite capping layer onto the film.^27,30^ A subsequent thermal treatment at *T* = 100 °C is performed to remove the remaining solvent inside the pore network and allow the perovskite material (CsPbBr_3_ or Cs_4_PbBr_6_) to crystallize. Figure 1b shows a SEM cross-section of one of the so obtained films. Only nanophosphor particles are clearly visible at this magnification, as PNCs located within the pores are expected to be present in low concentrations (5%–10% v/v according to inductively coupled plasma measurements) and have an average size of 5 nm, in the case of CsPbBr_3_, or form a continuous nanocoating onto the pore walls, in the case of Cs_4_PbBr_6_.^28,31^Figure 1c shows the X-ray diffraction (XRD) patterns of CsPbBr_3_ NCs embedded in GdVO_4_:Eu^3+^, Dy^3+^ phosphor nanoparticle film (CsPbBr_3_@GdVO_4_:Eu^3+^/Dy^3+^, green curve) and Cs_4_PbBr_6_@GdVO_4_:Dy^3+^ (violet curve). The crystalline structures of the phosphor nanoparticles (black or gray lines, PDF-01-086-0996), CsPbBr_3_ (green line, PDF-01-084-0464) and Cs_4_PbBr_6_ (violet line, PDF-01-073-2478) can be readily assigned. Nanophosphors show the most intense diffraction peaks, as they are the main component of the film. More specifically, this analysis confirms that GdVO_4_ nanoparticles grow in a single tetragonal host phase and feature a crystallite size of ∼35 nm, regardless of the doping ion. Also, diffraction peaks at 2θ = 15.3, 21.7, 30.7, 37.8 and 43.8° can be unambiguously assigned to CsPbBr_3_ orthorhombic phase, and peaks at 2θ = 12.8 and 29.7° to rhombohedral Cs_4_PbBr_6_, which confirms the presence of the perovskite compounds within the phosphor nanoparticle films.

### Optical Properties of Perovskite/Phosphor Hybrid Films

**Figure 2:**
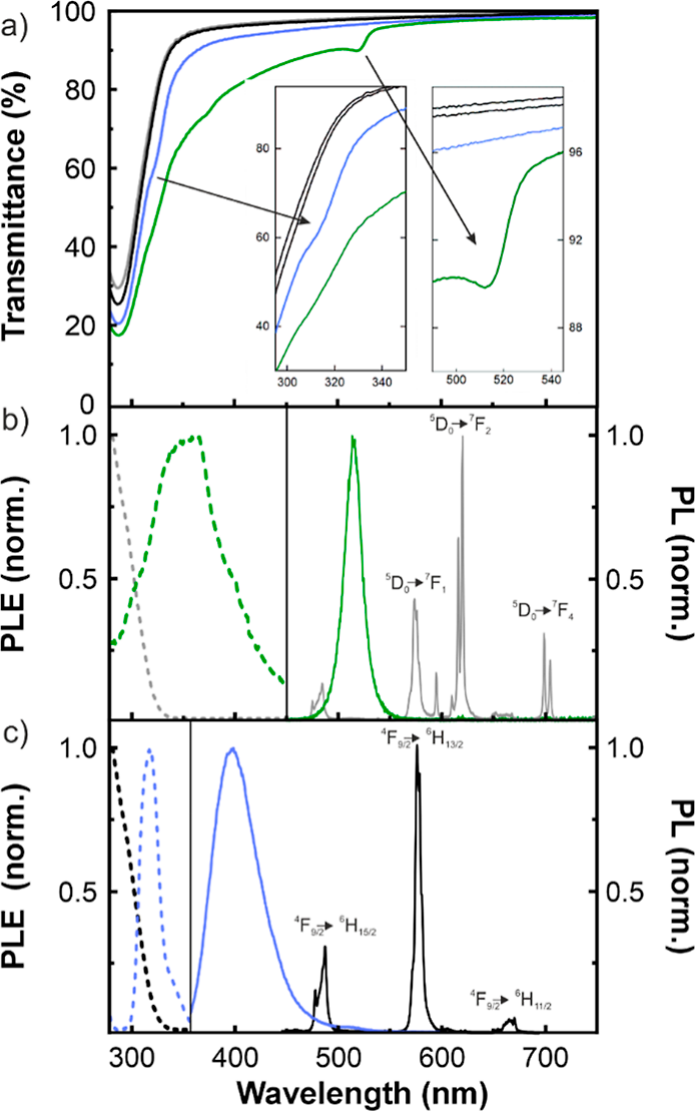
(a) Ballistic transmittance spectra of GdVO_4_:Eu^3+^/Dy^3+^ (gray), GdVO_4_:Dy^3+^ (black), CsPbBr_3_@GdVO_4_:Eu^3+^/Dy^3+^ (green) and Cs_4_PbBr_6_@GdVO_4_:Dy^3+^ (violet) films. The inset shows a detail of the spectral fingerprint of perovskite nanomaterials. (b) The left panel shows the photoluminescence (PL) excitation spectra of the CsPbBr_3_@GdVO_4_:Eu^3+^/Dy^3+^ film monitored at 620 nm (gray dashed line) and 515 nm (green dashed line). The right panel shows the PL spectra of the same film when the excitation wavelength is 280 nm (solid gray line) and 400 nm (solid green line). (c) The left panel shows the PL excitation spectra of the Cs_4_PbBr_6_@GdVO_4_: Dy^3+^ film monitored at 580 nm (black dashed line) and 400 nm (violet dashed line). The right panel shows the PL spectra of the same film when the excitation wavelength is 280 nm (solid black line) and 350 nm (solid violet line). Electronic transitions associated with the emission peaks of the Dy^3+^ and Eu^3+^ cations are indicated as labels.

Ballistic transmittance (*T*) measurements, as shown in Figure 2a, indicate that the bare nanophosphor films have values above 90% throughout the visible spectrum (see black line and Figure S2), highlighting the high transparency of the GdVO_4_ nanoparticle scaffolds (average visible transparency, AVT, of 98%). A decrease in the transmittance of the CsPbBr_3_@GdVO_4_:Eu^3+^/Dy^3+^ film is observed (green curve) for energies above the perovskite bandgap, below λ ≈ 510 nm. Consistently, the absorption band edge observed for the Cs_4_PbBr_6_@GdVO_4_:Dy^3+^ film (blue curve) presents a shoulder at 315 nm associated with the absorption of Cs_4_PbBr_6_.^47^ In both cases, the photoluminescence (PL) of perovskite and phosphor nanomaterials present in the films may be separately excited by using different excitation wavelengths (λ_ex_). Thus, the right panel of Figure 2b shows the PL spectra resulting of irradiating the CsPbBr_3_@GdVO_4_:Eu^3+^/Dy^3+^ film with λ_ex_ = 280 nm (gray curve) and λ_ex_ = 400 nm (green curve), which selectively excite the emission of the phosphor host or the perovskite guest, respectively. The former is readily identified by the narrow lines characteristic of the electronic transitions of Eu^3+^ (^5^D_0_ → ^7^F_1_, ^5^D_0_ → ^7^F_2_, and ^5^D_0_ → ^7^F_4_) and the Dy^3+^ (^4^F_9/2_ → ^6^H_15/2_, ^4^F_9/2_ → ^6^H_13/2_, and ^4^F_9/2_ → ^6^H_11/2_), while the later shows a distinctly broader green emission at λ = 515 nm corresponding to a quantum confined excitonic transition in CsPbBr_3_. Accordingly, PL excitation (PLE) spectra attained from the same film at the characteristic emission wavelengths of each component are also very different. The left panel in Figure 2b shows the result of monitoring the emission intensity at either λ = 620 nm (an intense Eu^3+^ line) or λ = 515 nm (peak of the blue-shifted CsPbBr_3_ band) as gray and green dashed curves, respectively. Similar effects are observed, and displayed in Figure 2c, for Cs_4_PbBr_6_@GdVO_4_:Dy^3+^ films. In this case, upon selective excitation at λ_ex_ = 315 nm and λ_ex_ = 280 nm, respectively, the Cs_4_PbBr_6_ emits a broad blue band (blue curve) and the host the narrow lines of Dy^3+^ (black curve). Like in the previous case, the PLE spectrum of the film has a strong dependence on the monitored emission wavelength, as it can be seen in the black and blue dashed curves plotted in the left panel of Figure 2c, corresponding to either λ = 580 nm (a Dy^3+^ line) or λ = 400 nm (characteristic of Cs_4_PbBr_6_),^47^ respectively. Interestingly, the PLE analysis of both types of films shows that there is a spectral range for which the phosphor host and the perovskite guest are simultaneously and efficiently excited. The potential of this effect to attain chromatically tunable emission from transparent films is discussed next.

### Coatings of Tunable Color: Warm and Neutral White Hues

**Figure 3:**
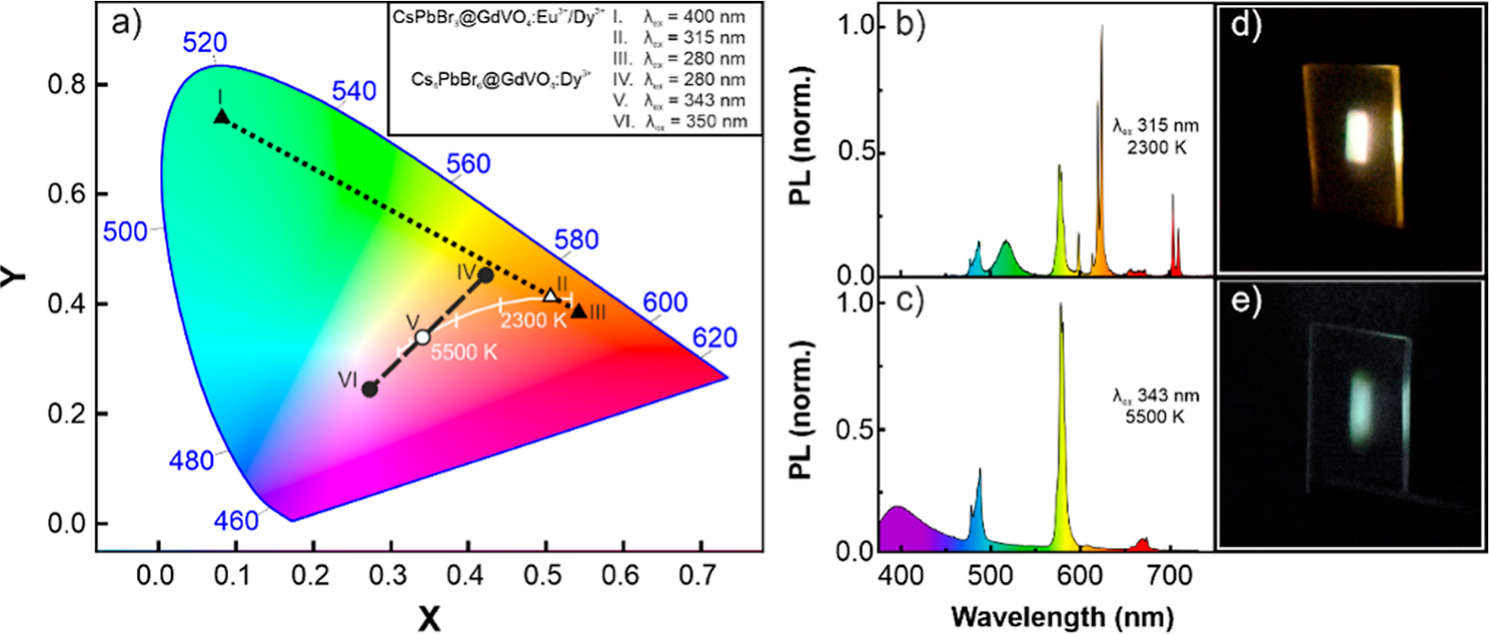
(a) CIE 1931 chromaticity coordinate of the light emitted by the CsPbBr_3_@GdVO_4_:Eu^3+^/Dy^3+^ and the Cs_4_PbBr_6_@GdVO_4_:Dy^3+^ films as a function of the photoexcitation wavelength. The white line indicates the Planckian locus. (b–e) PL spectrum of the CsPbBr_3_@GdVO_4_:Eu^3+^/Dy^3+^ film excited with 315 nm light (b) and the Cs_4_PbBr_6_@GdVO_4_:Dy^3+^ film with 343 nm light (c). Images of the white light emission of such films is shown in (d) and (e), respectively.

We explore the photoexcitation dependence of the emission by analyzing its chromaticity coordinates as a function of λ_ex_, as plotted in the CIE 1931 color space in Figure 3a. The photoexcitation of the CsPbBr_3_@GdVO_4_:Eu^3+^/Dy^3+^ coating with λ_ex_ = 400 nm yields the green emission associated with the CsPbBr_3_ nanocrystals (see point I in Figure 3a), while upon illumination with λ_ex_ = 280 nm, the emission of the coating corresponds to that of the mixed Dy^3+^ and Eu^3+^ doped phosphor nanoparticles of the scaffold (see point III in Figure 3a). By gradually sweeping λ_ex_ between these two wavelengths, different chromaticity values may be controllably attained (see Figure S3). This is a direct consequence of the different PL excitation spectra associated with each component of the coating (as shown in Figure 2b). As λ_ex_ red-shifts above 280 nm, the absorption cross section of the phosphors decreases and the contribution of the CsPbBr_3_ nanocrystals to the overall emission of the coating increases. As a result, a variety of hues from orange to green can be obtained. This includes white light, which is emitted when the CsPbBr_3_@GdVO_4_:Eu^3+^/Dy^3+^ layer is specifically photoexcited at λ_ex_ = 315 nm (see point II in Figure 3a). Figure 3b shows the corresponding PL spectrum with a balanced contribution from both perovskite and phosphor emitters. Note that the distinct presence of Eu^3+^ emission bands in the red part of the spectrum is key to achieving white light with low correlated color temperature. Similarly, when the Cs_4_PbBr_6_@GdVO_4_:Dy^3+^ film is photoexcited at λ_ex_ = 280 nm, a yellowish emission associated with the nanophosphor-based scaffold is obtained, whereas at λ_ex_ = 350 nm, a light blue emission is observed (see points IV and VI, respectively, in Figure 3a), which is due to the emission of Cs_4_PbBr_6_ nanocrystals and GdVO_4_:Dy^3+^ nanoparticles spectrally overlapping at 480 nm (see Figure 2c). By fine-tuning λ_ex_, it is possible to demonstrate any emission with color coordinates within the dashed line connecting such 2 points in the color space (see also Figure S4). Interestingly, choosing λ_ex_ = 343 nm results in a neutral white light (5500 K) whose PL spectrum is shown in Figure 3c, where the different contributions of the characteristic narrow lines of the Dy^3+^ cations and the broadband emission of the perovskite nanocrystals can be clearly identified. Note that the PLQY of these films is about 5% at the excitation wavelengths that produce white light. In addition, images of the white light emitted by the coatings are shown in Figure 3d,e. Finally, photographs and their chromatic coordinates of the points I to VI are presented in Supporting Information (Figure S5 and Table S1).

Finally, we analyzed the stability of the emission. It is noteworthy that the porous matrix improves the stability of the perovskite nanomaterials, preventing degradation under air or moisture (see Figure S6), in contrast to homogeneous layers of perovskite materials, which are very sensitive to moisture and oxygen in the environment.^30^ Therefore, our results confirm the multifunctional character of the nanophosphor scaffold, as it also acts as a physical protective barrier against the surrounding atmosphere, preserving the optical properties of the embedded perovskite nanomaterials.

### Conclusions

We have demonstrated a simple way to obtain tunable hue white light emission from transparent films using perovskite nanomaterials embedded in scaffolds of phosphor nanoparticles, enabling warm (2300 K) and neutral (5500 K) hues selectively controlled by the applied excitation wavelength. Our approach is based on the use of GdVO_4_:Eu^3+^ and GdVO_4_:Dy^3+^ phosphor nanoparticles, which can be processed as porous thin films that act as transparent multifunctional scaffolds since they do not scatter light and serve as nanoreactors for the synthesis of CsPbBr_3_ and Cs_4_PbBr_6_ nanocrystals, while also emitting light when photoexcited in the UV. Interestingly, the scaffold also provides the nanomaterials with enhanced stability against air and moisture. A careful mixing of the phosphor nanoparticle emission with that of the perovskite nanocrystals can be achieved by adjusting the photoexcitation wavelength without changing the chemical composition of the emitters. Our results provide an alternative route for the development of stable perovskite emitters with broadband spectra of interest in the context of illumination, sensing or signaling.

## Materials and Methods

### Materials

Gadolinium(III)nitrate hexahydrate [Gd(NO_3_)_3_·6H_2_O, Aldrich 99.9%], europium(III)nitrate pentahydrate [Eu(NO_3_)_3_·5H_2_O, Aldrich, 99%], dysprosium(III)nitrate hydrate [Dy(NO_3_)_3_·*x*H_2_O, Aldrich, 99%], sodium orthovanadate (Na_3_VO_4_, Aldrich, 99.9%), PAA (average Mw 1800, Aldrich), ethylene glycol, absolute ethanol, methanol, Milli-Q water, cesium bromide (CsBr, TCI, 99,9%), lead(II) bromide (PbBr_2_, TCI, 99.9%), dimethyl sulfoxide (DMSO, Merck, anhydrous, 99.8%) were used as received.

### Preparation of Phosphor Nanoparticle Thin Film

GdVO_4_:Eu^3+^ and GdVO_4_:Dy^3+^ nanophosphors were synthesized following a solvothermal synthesis reported elsewhere.^43,45^ Nanoparticles were deposited over the substrate via spin-coating under the following conditions: two depositions at 2000 rpm for 1 min. Finally, the film was annealed at 400 °C for 30 min.

### Perovskite Synthesis in Nanophosphor Porous Matrix

CsPbBr_3_ solution was prepared dissolving CsBr and PbBr_2_ in a 106 mM concentration in DMSO while Cs_4_PbBr_6_ solution was prepared dissolving CsBr in a 106 mM and PbBr_2_ in a 26.5 mM concentration in DMSO. Both solutions were spin-coated (Ossila spin-coater) on top of a phosphor nanoparticle film at 5000 rpm for 1 min with a 5000 rpm/s acceleration followed by an annealing step at 100 °C during 60 min. These processes were performed inside a nitrogen-filled glovebox.

### Characterization

Cross-section micrograph of nanophosphor particle films was obtained by scanning electron microscopy (SEM, Hitachi model S4800 high-resolution microscope) using a 2 kV voltage and a 10 μA current. X-ray diffractograms were collected in a X’pert PRO X-ray diffractometer (Philips) using Cu Kα radiation (λ = 1.54518 Å) in 5–60° 2θ range with 0.05° step under a grazing incidence (angle of incidence θ = 1°) Total transmittance of films was obtained using a UV–vis–NIR spectrophotometer (Cary 5000, Agilent) with a DRA-2500 (PMT/PbS) detector with incident light on samples at ∼6°. Photoluminescence and photoluminescence excitation spectra were acquired using a spectrofluorometer (FLS1000, Edinburgh instruments) where white light is generated by a 450 W ozone-free Xenon arc lamp and excitation wavelength is set with a monochromator. Stability experiments were performed by placing the samples in a homemade closed chamber with quartz windows in which humidity is controlled by independently adjusting the flow of wet and dry nitrogen gas. The chamber was specifically designed to be mounted in the spectrofluorometer bench.

## Data Availability

The data supporting the results of this study are openly available in the Digital CSIC repository at https://doi.org/10.20350/digitalCSIC/17159.

## Supporting Information Available

Structural characterization, CIE color coordinates, stability measurements (PDF)
