# Supplementary material for: Tunable White Light Emission from Transparent Nanophosphor Films Embedding Perovskite Lead Halide Nanostructures
Source: ACS Appl Mater Interfaces. 2025 Mar 19;17(13):19900–5. doi: 10.1021/acsami.4c22044 (PMC11969428; doi:10.1021/acsami.4c22044)
Supplement: Supplementary file 1 — am4c22044_si_001.pdf [file am4c22044_si_001.pdf]

Supporting Information of

Tunable White Light Emission from Transparent Nanophosphor Films Embedding Perovskite Lead Halide Nanostructures

*AUTHOR NAMES: José María Viaña, ‡ Carlos Romero-Pérez, ‡ Mauricio E. Calvo *, Gabriel Lozano*, Hernán Míguez.*

AUTHOR ADDRESS: Instituto de Ciencia de Materiales de Sevilla, Consejo Superior de Investigaciones Científicas-Universidad de Sevilla, Calle Américo Vespucio 49, 41092 Sevilla, Spain.

*E-mail: mauricio.calvo@csic.es, g.lozano@csic.es

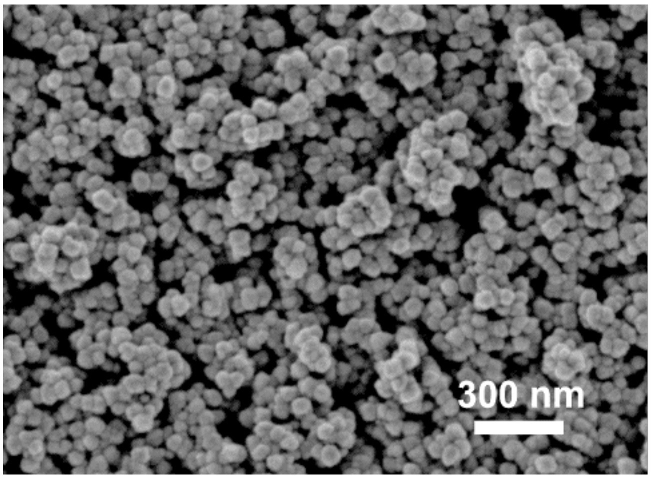

Figure S1. *SEM top-view micrograph of GdVO4: Eu3+/Dy3+ film employed in perovskite synthesis (24.5k magnification).*

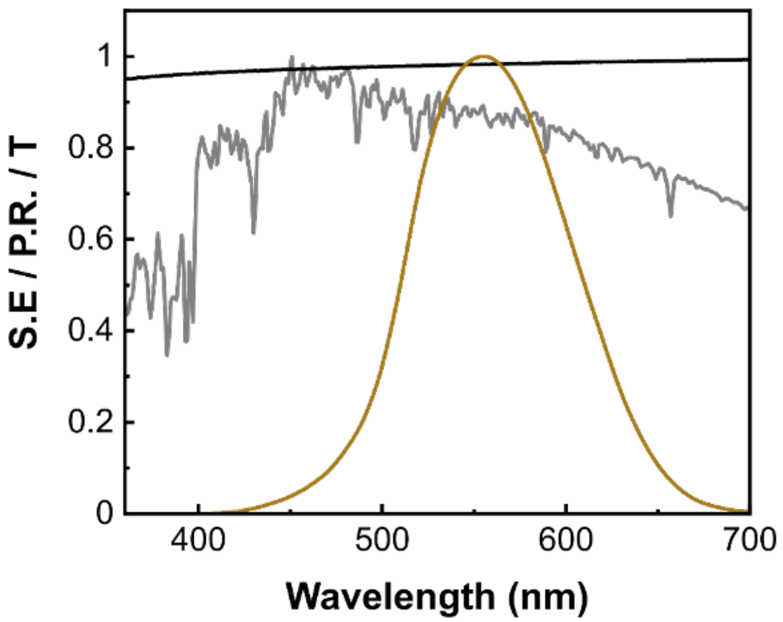

Figure S2. *AM 1.5G solar photon flux (S.E.; gray), photopic response (P.R.; light brown) and film transmittance (T; black) spectra used for Average Visible Transparency calculation. Please notice all spectra are normalized in this representation.*

$$AVT = \frac{\int T(\lambda) \cdot V(\lambda) \cdot \mathrm{AM1.5G}(\lambda)}{\int V(\lambda) \cdot \mathrm{AM1.5G}(\lambda)}$$

Equation S1. Average Visible Transmittance (AVT) calculation where T(λ) is the transmittance, V(λ) is the photopic response and AM1.5G(λ) is the standard solar spectral irradiance.

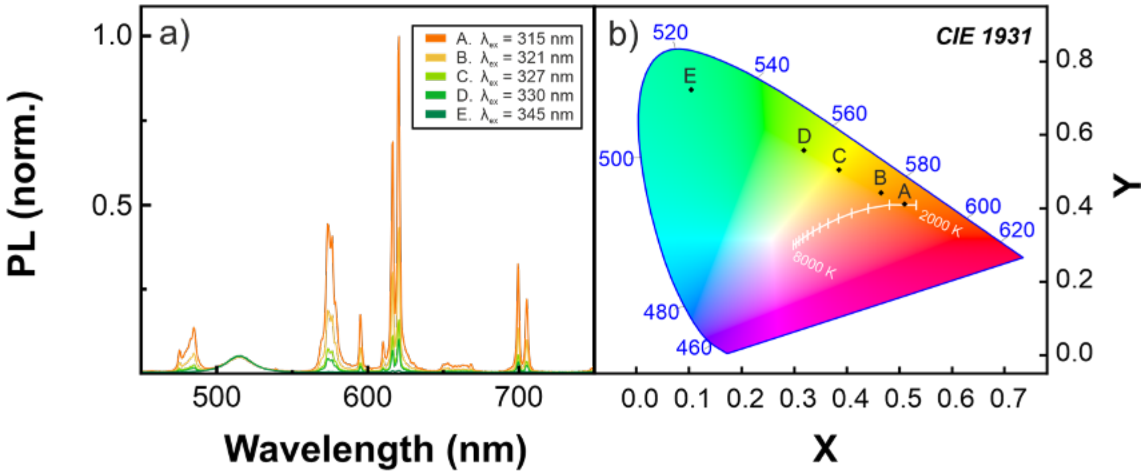

Figure S3 *CsPbBr$_3$@GdVO$_4$:Eu^{3+}/Dy^{3+} photoluminescence spectra under an excitation wavelength scan (a) and their corresponding excitation-dependent chromatic tunability (b).*

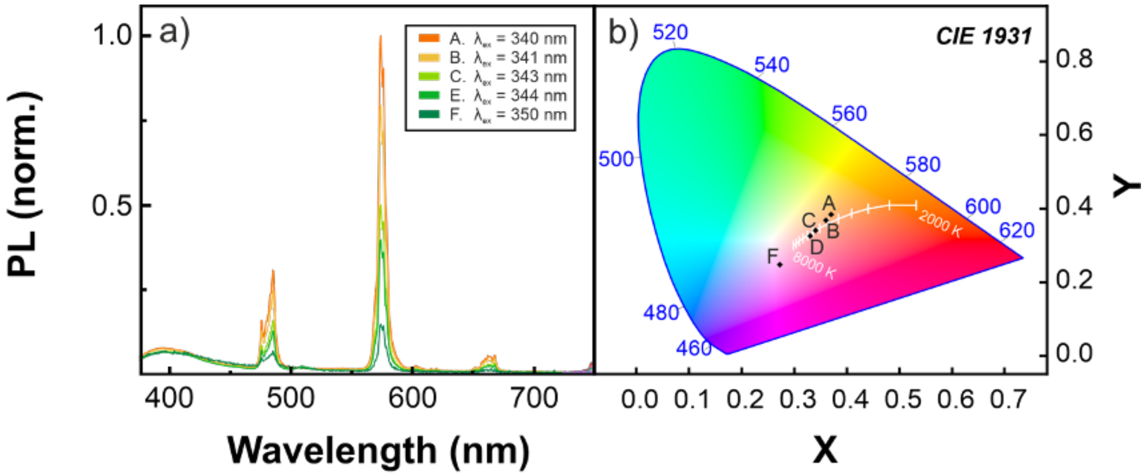

Figure S4*. Cs$_4$PbBr$_6$@GdVO$_4$:Dy^{3+} photoluminescence spectra under an excitation wavelength scan (a) and their corresponding excitation-dependent chromatic tunability (b).*

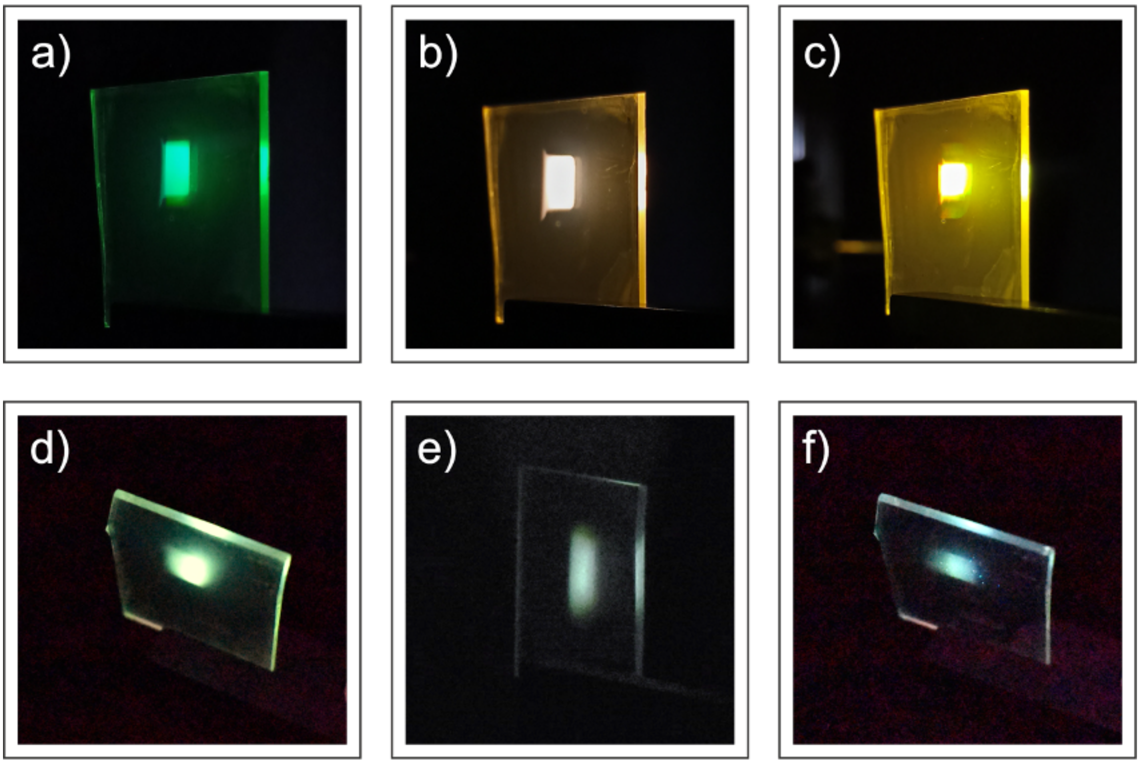

Figure S5. *Digital pictures of $CsPbBr_3@GdVO_4:Eu^{3+}/Dy^{3+}$ and $Cs_4PbBr_6@GdVO_4:Dy^{3+}$ under different excitation wavelengths: $CsPbBr_3@GdVO_4:Eu^{3+}/Dy^{3+}$ λ_{ex} = 400 nm (a), $CsPbBr_3@GdVO_4:Eu^{3+}/Dy^{3+}$ λ_{ex} = 315 nm (b), $CsPbBr_3@GdVO_4:Eu^{3+}/Dy^{3+}$ λ_{ex} = 280 nm (c), $Cs_4PbBr_6@GdVO_4:Dy^{3+}$ λ_{ex} = 280 nm (d), $Cs_4PbBr_6@GdVO_4:Dy^{3+}$ λ_{ex} = 343 nm (e) and $Cs_4PbBr_6@GdVO_4:Dy^{3+}$ λ_{ex} = 350 nm (f).*

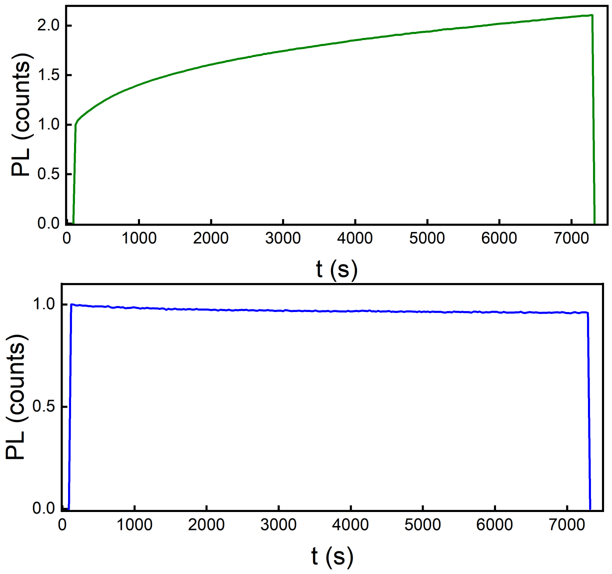

Figure S6. *PL intensity normalized at t=120s recorded at the wavelength of the maximum emission of $CsPbBr_3$ (top, green) and Cs_4PbBr_6 (bottom, violet) nanomaterials embedded in a nanophosphor scaffold under constant illumination at λ_{ex}=315nm and λ_{ex}=343nm, respectively, at relative humidity RH= 50%..*

Material	λ_{ex} (nm)	CIE1931 coordinates (x, y)
$CsPbBr_3$@$GdVO_4$:Eu^{3+}/Dy^{3+}	400	0.0775, 0.7395
$CsPbBr_3$@$GdVO_4$:Eu^{3+}/Dy^{3+}	315	0.5094, 0.4114
$CsPbBr_3$@$GdVO_4$:Eu^{3+}/Dy^{3+}	280	0.5423, 0.3861
Cs_4PbBr_6@$GdVO_4$:Dy^{3+}	280	0.4057, 0.4524
Cs_4PbBr_6@$GdVO_4$:Dy^{3+}	343	0.3338, 0.3486
Cs_4PbBr_6@$GdVO_4$:Dy^{3+}	350	0.2698, 0.2521

Table S1. CIE1931 coordinates of the photoluminescence of $CsPbBr_3$@$GdVO_4$:Eu^{3+}/Dy^{3+} and Cs_4PbBr_6@$GdVO_4$:Dy^{3+} under different excitation wavelengths
